# Records Boston Medical Society

**Published:** 1855-05

**Authors:** R. M. Hodges

**Affiliations:** Secretary


### FROM THE RECORDS OF BOSTON SOCIETY FOR MEDICAL OBSERVATION.

BY R. M. HODGES, M. D., SECRETARY.

Bleeding in Pneumonia.—Dr. Williams, in the course of a discussion upon the treatment of pneumonia, said that in Vienna the expectant treatment had been found as successful in pneumonia as active treatment and blood-letting. He also mentioned that an old and intelligent country physician had made the remark to him that, according to his observations, the type of pneumonia had entirely changed during the last forty years, and that with this change he had been obliged to change his treatment; whereas he used always to bleed, now he never did.

Dr. Slade said that Dr. Stokes, of Dublin, had not bled in pneumonia for twenty years.

In reply to a question, Dr. Bowditch said that the treatment at the Massachusetts General Hospital was according to circumstances, viz., active treatment, bleeding and antimonials; or expectant, simply.

Dr. Buckingham mentioned a case of pneumonia and pleurisy, in which the "let alone treatment" had been pursued, and which lasted from the middle of August to the first of October. Depletory measures could not be made use of. The pneumonic symptoms lasted four weeks. No tubercular diathesis or predisposition. Dr. B. objects to counter-irritation in acute thoracic disease, never having been satisfied as to its beneficial influence.

Seminal Emissions.—Dr. Cabot had used lupuline, in half-drachm doses two or three times a-day, with good success in cases of seminal emissions. Cold baths taken merely as a 'dip' he thought did harm, but when prolonged sufficiently to chill the parts were certainly beneficial.

Dr. Buckingham had seen patients who tried to cure themselves by a strict regimen of diet and sleep, but he had always advised an opposite course. In his cases oxalate of lime had usually been found in the urine, and such patients had been relieved by citrate of iron. In patients worn down by excessive venery, he had found strychnia useful. He usually advised the removal of books on the subject of onanism, and did not recommend coitus or the avoiding of female society. The "spermatorrhœa ring" he thought more likely to produce seminal emissions, by irritation, than to cure them.

Dr. Slade thought the "ring" sure to arrest the emissions, if kept on. Intercourse, to be of service, must be regular. Oxalate of lime found in these cases was owing to the state of the patient's general health and the affection of the brain which accompanied it.

Dr. Bacon said that Donne had thought the presence of oxalate of lime in the urine diagnostic of seminal emissions. This was well known now not to be the fact, but to depend, as Dr. Slade has said, upon the condition of general health, and the nervous derangement of the patient. The deposit is most frequently found after changes of temperature in the summer season.

Dr. Buckingham said that in the last two cases where he had observed oxalate of lime in the urine in connection with seminal emissions, the patients were stout and robust. Their attention had been recently called to their condition, but it was accompanied by no depression of nervous energy. Dr. B. mentioned a case where zoosperms had been found in the urine of a woman 8 months advanced in pregnancy, and thought that half the time when found in the urine they were the result of a natural evacuation.

Dr. Williams said that in a case of his, relieved by a combination of lupuline with ergot, the patient was an officer in the army and took a very rational view of his case. He was consulted as to the expediency of cauterization. He did not recommend it. Strychnia had been tried without success, but the lupuline and ergot were effectual.

SUDDEN FORMATION OF CATARACT.—Dr Cabot mentioned the following instance: A girl, 16 years old, was operated upon by him for strabismus. The result of the operation was good, and the vision perfect for four days after. In the night of the fourth day she had great pain in her eye-ball, and woke up on the fifth day with well-marked cataract. She was of a scrofulous diathesis.

LEMON JUICE AS A SEDATIVE TO THE PAIN CAUSED BY PASSAGE OF BILIARY CALCULI.—Dr. Bowditch had used this with great success in a case under his care. Formerly the paroxysms of pain lasted a day or two; since its exhibition the patient has none at all. He supposed the action to be similar to that of the nitro-muriatic acid bath. The similarity between the symptoms of duodenitis and those of biliary calculi was remarked upon, and the relief which lemon juice causes in that disease was alluded to.

PECULIAR EFFECT OF CHLOROFORM.—Dr. E. H. Clarke mentioned this case, which occurred in the practice of another physician. A girl, 20 years old, inhaled chloroform for the purpose of having a tooth extracted. She recovered apparently from its influence, and walked home the distance of a quarter of a mile.—Her conversation was however incoherent, and her gait unsteady. Soon after reaching home she became paralyzed, losing both sensation and the power of motion. The skin was cold and pale; respiration *saccadic* and the pulse feeble; no rigidity of the muscles. She came out of this state, and then became furiously insane, together with which were constipation and deficient secretion of urine. This condition of things lasted from a week to ten days, and then her usual health returned.

Clay-colored Fæces without deficiency in the Biliary Secretions.—Dr. Ellis mentioned an instance where the discharges were clay-colored for some time previous to death, and at the autopsy the fæces in the upper part of the intestine were yellow, and in the lower part white, showing that the secretions of the intestine are as necessary to give the natural fæcal color as the bile itself. Dr. E. remarked upon the importance of this fact, as in such cases it is the liver that is always blamed, when very possibly it may be the intestines that are at fault.—*Boston Med. Jour.*

